# Theoretical study of the electronic and optical properties of a composite formed by the zeolite NaA and a magnetite cluster

**DOI:** 10.3762/bjnano.16.5

**Published:** 2025-01-17

**Authors:** Joel Antúnez-García, Roberto Núñez-González, Vitalii Petranovskii, H’Linh Hmok, Armando Reyes-Serrato, Fabian N Murrieta-Rico, Mufei Xiao, Jonathan Zamora

**Affiliations:** 1 Facultad de Ciencias, Universidad Autónoma de Baja California, Carretera Transp. Ensenada-Tijuana, Fracc. Playitas No. 3917, Ensenada, B. C. 22860, México; 2 Departamento de Matemáticas, Universidad de Sonora, Blvd. Luis Encinas y Rosales s/n, 83000, Hermosillo, Sonora, México; 3 Universidad Nacional Autónoma de México, Centro de Nanociencias y Nanotecnología, 22800 Ensenada, B. C., México; 4 Simulation in Materials Science Research Group, Science and Technology Advanced Institute, Van Lang University, Ho Chi Minh City, Vietnam; 5 Faculty of Applied Technology, School of Technology, Van Lang University, Ho Chi Minh City, Vietnam; 6 Universidad Politécnica de Baja California, Ingeniería Mecatrónica, Mexicali, Baja California 21376, México; 7 Instituto Potosino de Investigación Científica y Tecnológica A.C., Camino a la Presa San José 2055, Col. Lomas 4, San Luis Potosí, S.L.P. 78216, México

**Keywords:** magnetic cluster, NaA zeolite, optical properties

## Abstract

The electronic and optical properties of a composite created by introducing a magnetite cluster into NaA zeolite have been investigated in this work using DFT calculations. The results obtained indicate that the electronic and optical properties of the composite are enhanced because of the cluster. However, the properties exhibited by the cluster outside the zeolite differ from those it presents when it is part of the composite. It is noteworthy that the composite exhibits magnetic properties of a half-semiconductor and a strong optical response within the visible and ultraviolet regions of the spectrum.

## Introduction

Zeolites are crystalline materials made up of aluminosilicates with a three-dimensional structure comprising pores and cavities of molecular dimensions. This unique structure enables them to operate as molecular sieves, allowing molecules smaller than the pore size to pass through while blocking the diffusion of larger ones. Furthermore, the physicochemical characteristics of zeolites depend largely on the chemical composition of the framework, specifically the Si/Al ratio [1–2]. These characteristics make zeolites highly appealing for a wide range of applications, including the production of fine chemicals [3–4], gas separation [5–7], ion exchange [8–10], heavy metal removal [11–12], sensor technologies [13–16], and biomedical applications [17].

Nanoscale materials represent a thriving field of research with a wide range of potential applications. Today, it is generally recognized that properties like hardness, reactivity, toxicity, and optical response are intricately linked to factors such as the chemical composition, particle size, structure, and geometry of these materials [18–20]. Hence, it is generally undesirable for nanoscale materials to undergo structural alterations because of environmental exposure or to change their properties because of the migration and coalescence of nanoparticles on the carrier material [21–22]. Such changes can significantly modify the physicochemical properties of the original nanomaterial. Also, the most interesting physicochemical properties are exhibited by clusters with subnanometer dimensions. For example, the active centers of the most efficient heterogeneous catalysts commonly fall within this range [23–26]. The challenge lies in the fact that, because of their pronounced tendency to aggregate, these materials must be deposited with a high level of dispersion to achieve the desired properties and performance. Furthermore, apart from not preventing potential exposure to unwanted molecules, the structural characteristics and, hence, the physicochemical properties of the cluster could be altered as a result of its interaction with the support material. Indeed, one viable solution to tackle these challenges is to utilize zeolites, which are frequently employed as inert support materials [27–32]. Zeolites are well suited for the hosting and confinement of molecular clusters with dimensions below 10 Å. This approach has the potential to stabilize these clusters and prolong their operational lifespan.

As zeolites are synthesized in powder form, they typically have grain sizes ranging from hundreds of nanometers to tens of micrometers. For applications where recovery at the end of a process is desirable, this can be a limitation. A very interesting alternative is the introduction of magnetic nanoparticles into zeolite crystals so that the resulting composite can respond to an external magnetic field [33]. By imparting magnetic properties to such composites, they can be efficiently recovered after capturing contaminants such as heavy metals [34–37] and dyes [38–40] in bodies of water, addressing a pressing environmental concern. Also, iron-modified zeolites have shown variations in both electric and magnetic properties that allow one to generate catalysts based on zeolites [41]. Among these types of composites consisting of zeolites modified with magnetic nanoparticles, sodium Linde A Type (LTA) zeolite, also known as NaA zeolite, stands out for its remarkable capacity and selectivity to capture various types of metals commonly found as contaminants in drinking water. These metals include Ca, K, Mg, Mn, Co, Zn, Cu, Pb, Cd, Cs, and Sr [42–46]. Because of the remarkable ion-exchange capacity of zeolites, their large surface area, and the well-organized porous systems with molecular sieve functionality, zeolites have long been fruitfully used in important industrial applications, mainly related to catalysis and wastewater treatment.

The rapid development of nanotechnology and the emergence of composite zeolite materials have opened up unprecedented opportunities for their application in nanomedicine [47]. The unique properties of magnetic nanoparticles allow them to be used for targeted drug delivery and visualization of internal organs [48]. Magnetic nanoparticles have unique magnetic properties and the ability to function at the cellular and molecular level of biological interactions. Of course, the evaluation of cytotoxicity and bioapplicability of each substance is a crucial issue before its use in clinical practice. Although there are fewer studies on the cytotoxicity of nanoparticles on zeolite carriers than other mesoporous matrices, most articles report low cytotoxicity of zeolites. Zeolites are classified as “Safe Substances for Food and Feed Additives” by the European Food Safety Authorities [49] and are “Generally Recognized as Safe” [50] by the United States Food and Drug Administration [51]. Also, iron-based magnetic compounds have the advantage of being a widely available and relatively cheap material, as well as being biocompatible and environmentally friendly [52–53].

While the potential of magnetic clusters to impart magnetic properties to zeolite composites is evident, a comprehensive understanding of these properties remains elusive because of the challenges in experimentally characterizing the structural properties of zeolite-hosted clusters. This field is relatively new, and experimental data on the precise structure and properties of these systems is very limited. Besides, to the best of our knowledge, there is a dearth of theoretical literature specifically addressing the study of magnetic clusters within zeolites. With this motivation, the present study evaluates the electronic properties of the magnetite cluster using DFT calculations and compares them to those in the case where the cluster is embedded within the NaA zeolite. Our work aims to provide insights into the structural and electronic properties of these systems, paving the way for future experimental investigations and the development of novel magnetic materials.

## Computational Details

**Figure 1:**
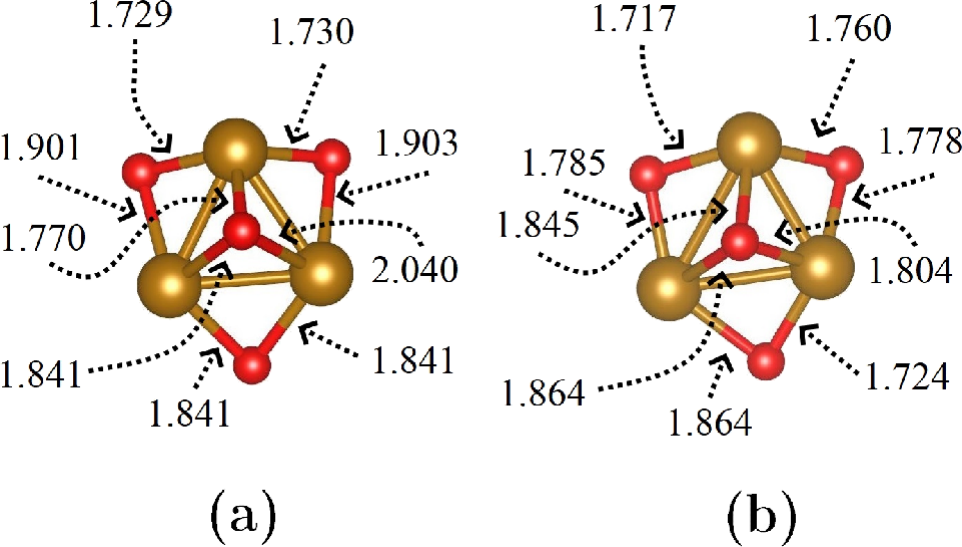
Geometry and Fe–O bond lengths for a magnetite cluster under two different conditions: (a) outside the NaA zeolite and (b) inside the NaA zeolite. Bond lengths are in angstroms.

**Figure 2:**
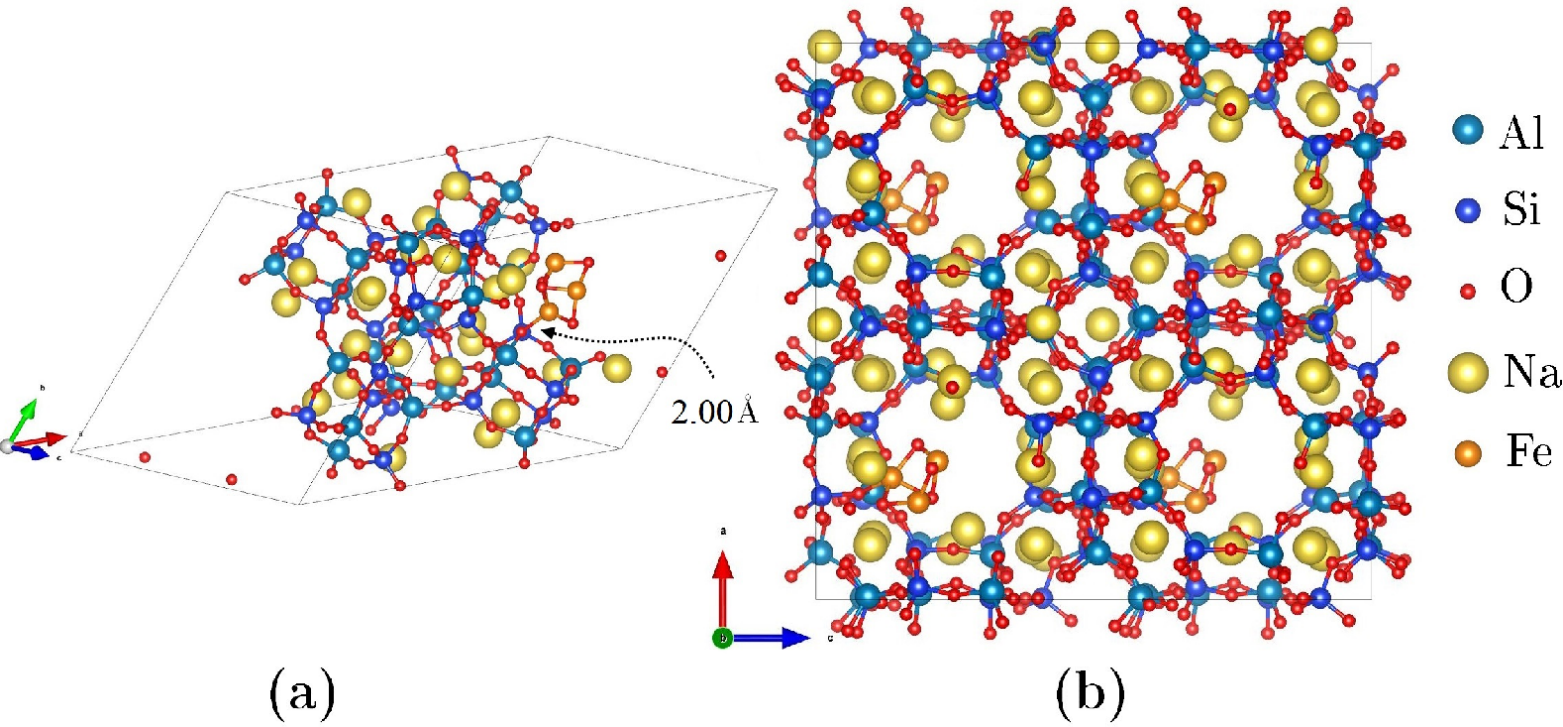
The optimized NaA-M composite is represented by two different types of cells: (a) a trigonal cell and (b) a cubic cell. The trigonal cell has two α-cages and one β-cage, whereas the cubic cell has eight α-cages and four β-cages. In both cell representations, magnetite clusters are hosted in only half of the α-cages. The red and brown spheres in both clusters represent oxygen and iron atoms, respectively.

In the current study, the dehydrated sodium LTA zeolite, commonly denoted as NaA in the literature, with a ratio of Si/Al = 1 was considered. For this purpose, we adopted the trigonal cell proposed by Antúnez-García et al. [54], for which the lattice parameters are *a* = *b* = *c* = 17.179 Å and α = β = γ = 60°, described by the chemical formula 12Na^+^[Al_12_Si_12_O_48_]^12−^. As an additional consideration, we will assume that the distribution of aluminum atoms in the framework satisfies Löwenstein’s rule [55]. Also, we considered the highly stable Fe_3_O_4_ magnetite minimal cluster (see Figure 1), as proposed by Ermakov et al. [56], for the purposes of this study. After separately obtaining the optical and electronic properties of both the NaA zeolite and the cluster, our next step involved locating the position of minimum energy for the cluster within the zeolite framework. In essence, we compared the energy difference (after optimization) between housing the cluster in the α-cage and the β-cage (see [54,57] for cage identification). The results demonstrated that housing the cluster in the α-cage is energetically more favorable (Figure 2). This choice defined the composite under investigation (labeled as NaA-M) and served as the basis from which we computed its optical and electronic properties in this study.

The electronic and optical properties of zeolite NaA, the magnetite cluster, and the NaA-M composite were computed using the Wien2k computer code [58]. This code employs the APW+lo method in conjunction with density functional theory (DFT) to calculate electronic structures. To determine the exchange–correlation interaction, we utilized the Tran–Blaha-modified Becke–Johnson (TB-mBJ) approximation. This approach provides calculated bandgap values that exhibit excellent agreement with experimental data [59]. In detail, TB-mBJ combines the modified Becke–Johnson exchange potential with the local density approximation (LDA) for the correlation potential. The parameters used for the calculations were the following: The muffin-tin radii *r*_mt_ are 1.70, 1.38, 1.60, 1.90, and 1.70 for aluminum, oxygen, silicon, sodium, and sulfur, respectively; the convergence number, that is, the smallest muffin-tin radii times the plane wave cutoff parameter, is set at *R*_mt_*K*_max_ = 6.0; the maximum *l* value for partial waves used inside atomic spheres is *l*_max_ = 10; and the magnitude of the largest vector in charge density Fourier expansion is *G*_max_ = 12.0. The energy to separate the valence states of the core states was set at a value of −7.5 Ry; thus, the Al [1s^2^ 2s^2^], O [1s^2^], Si [1s^2^ 2s^2^], Na [1s^2^], and Fe [1s^2^ 2s^2^ 2p^6^] electronic states are considered as core states, and the rest of electronic states as valence states. For integration in the reciprocal space, a 3 × 3 × 3 mesh (14 k-points in the irreducible Brillouin Zone (IBZ)) is used during the self-consistent cycle, and a 6 × 6 × 6 mesh (112 k-points in IBZ) for the calculation of density of states and optical properties. For the energy convergence criterion, we consider a value of 1 × 10^−4^ Ry. For a valid comparison, the same values were used to calculate the electronic and optical properties of the isolated magnetite cluster. Finally, spin polarization was considered for both calculations (zeolite-cluster and isolated cluster).

## Results and Discussion

In Figure 1a, the minimum energy configuration for the NaA-M composite is depicted, corresponding to the placement of the magnetite cluster within an α-cage of the NaA, rather than within a β-cage. In this figure, it can be observed that one of the Fe atoms from the magnetite cluster interacts with an oxygen atom from the pore surface, resulting in a Fe–O bond length of 2.00 Å. Figure 1b corresponds to the same composite but is described in a cubic cell, which shows clearly the location of the magnetite cluster in the α-cage. The representation of a composite in two different unit cells is possible because there exists a linear operator and its inverse that allow us to go from a trigonal to a cubic cell representation and vice versa. Figure 2a and Figure 2b display the structures and Fe–O bond distances of a magnetite cluster in both the isolated form and when it is introduced into the NaA zeolite to form the NaA-M composite. Comparing these structures directly and examining their respective Fe–O bond lengths reveal that the magnetite cluster undergoes structural changes when confined within the zeolite.

**Figure 3:**
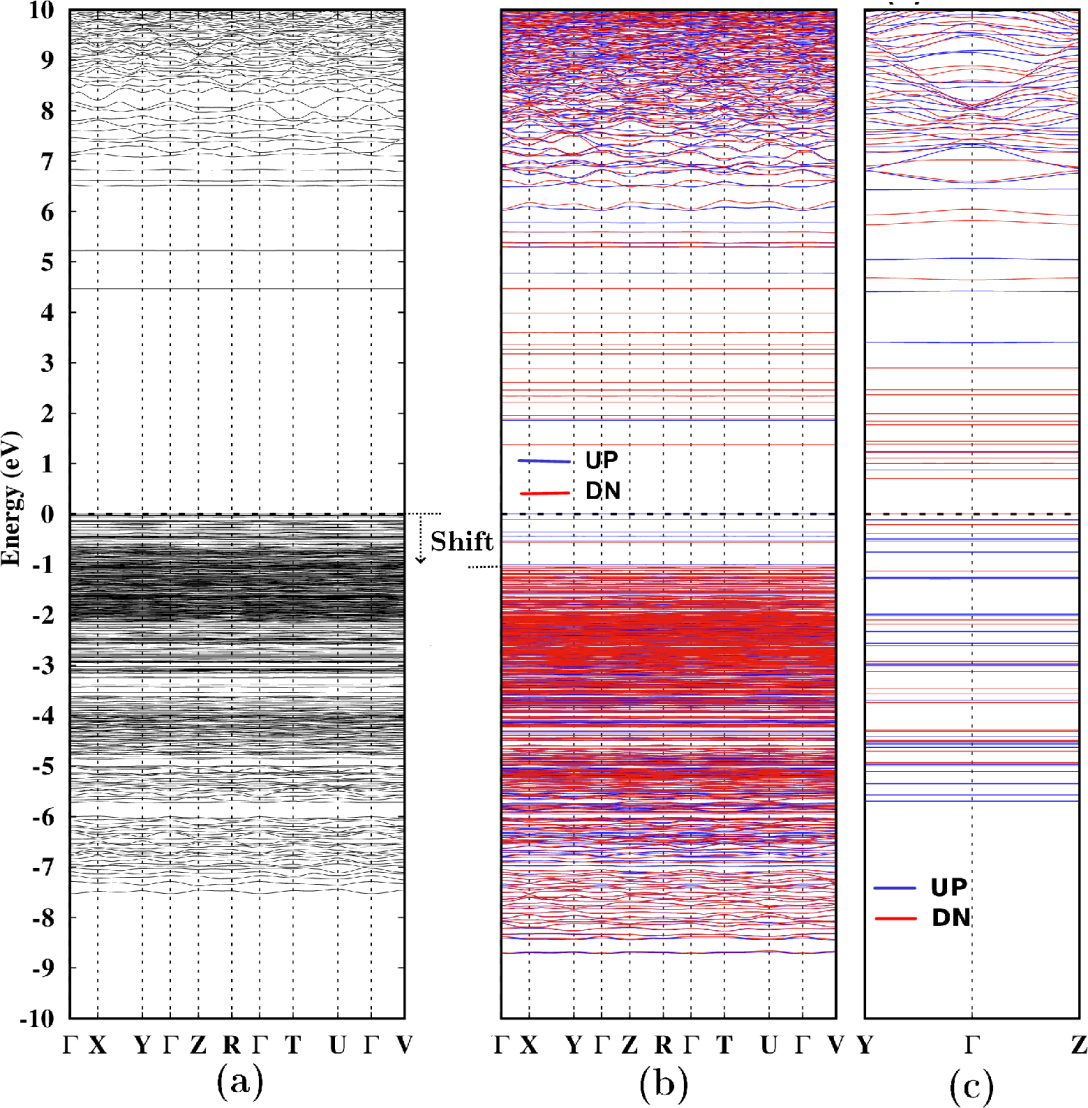
Band structure for (a) NaA zeolite, (b) NaA-M composite, and (c) isolated magnetite cluster. The blue and red bands distinguish the spin up and spin down states, respectively. Fermi level is located at 0 eV.

Figure 3a presents the band structure of the NaA zeolite, which exhibits no magnetic behavior. Notably, a pair of bands at 4.5 and 5.2 eV emerge within the forbidden zone. Previous research [60] has shown that these bands arise from the Na–O interaction and make a relatively low contribution to the total density of states (TDOS). In Figure 3b, when the magnetite cluster is introduced into the zeolite, new bands appear within the forbidden zone, and a decoupling of bands with spin up and spin down occurs, giving rise to magnetic behavior in the NaA-M composite. A comparison between Figure 3a and Figure 3b reveals that the introduction of the magnetite cluster reduces the bandgap and induces a shift in the band structure of the NaA zeolite toward negative energies, approximately by 1 eV. This observation is consistent with the results of prior research studies [61–63]. Figure 3c illustrates the band structure corresponding to the isolated magnetite cluster, focusing on the trajectory defined by the special points Y, Γ, and Z. A direct comparison between Figure 3b and Figure 3c highlights that the magnetite cluster introduces specific bands within the forbidden zone of the NaA zeolite. As it was expected, these bands do not entirely align with those of the isolated cluster. Given the observed structural modifications of the cluster when integrated into the zeolite (Figure 2), it is expected that its electronic properties would also undergo changes.

**Figure 4:**
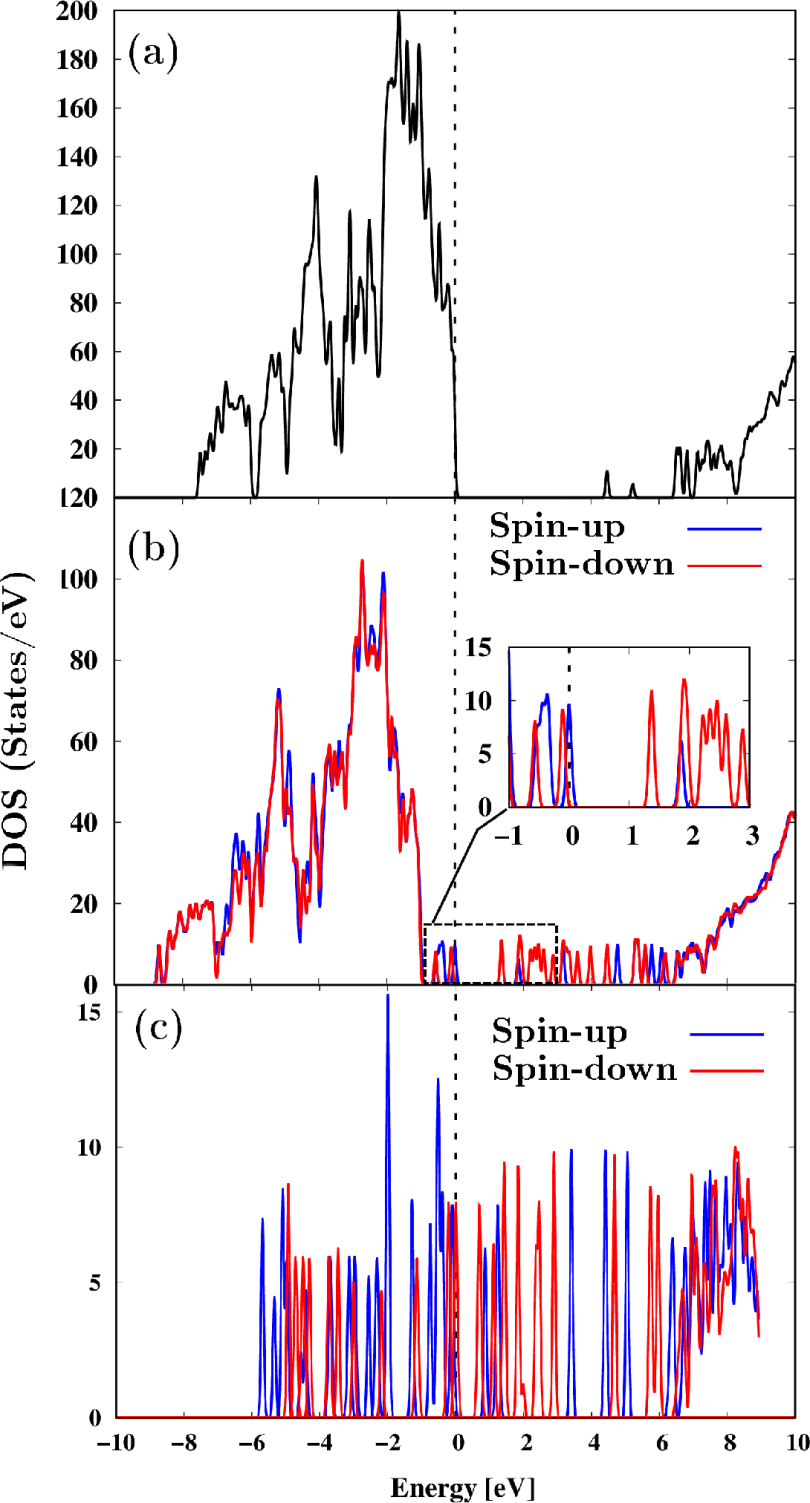
DOS for (a) NaA zeolite, (b) NaA-M composite, and (c) isolated magnetite cluster. Fermi level is located at 0 eV.

**Figure 5:**
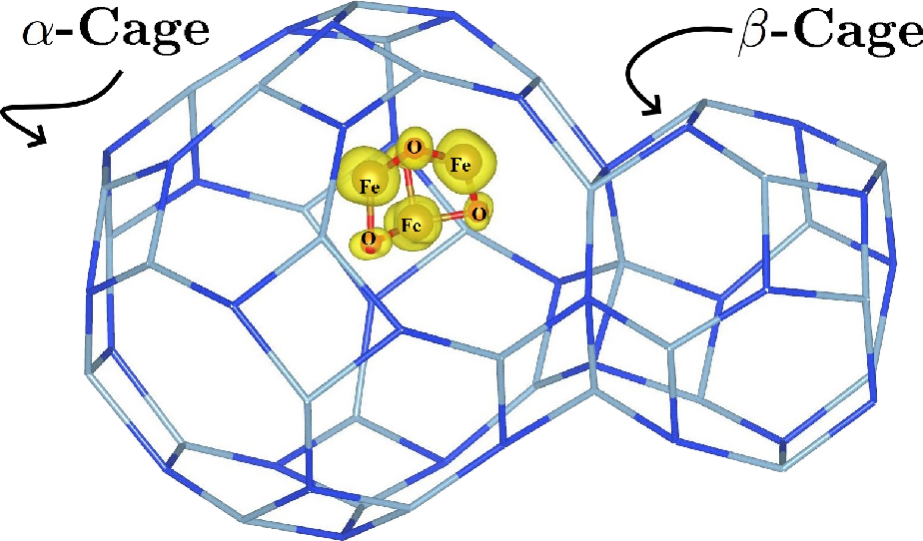
Spin density difference (up–down) isosurface of the NaA-M composite. For simplicity, the explicit depiction of the atoms constituting the zeolite framework has been omitted; instead, a wireframe representation has been employed.

Figure 4a displays the total density of states (TDOS) for the NaA zeolite, featuring a primary bandgap of 6.5 eV, along with additional bandgaps originating from states associated with the bands at 4.5 and 5.2 eV. In Figure 4b, the TDOS for the NaA-M composite is presented, clearly revealing the decoupling of the spin-up and spin-down states, resulting in a “half-semiconductor”-type magnetic behavior. In this figure, the enlarged region around the Fermi level highlights a distinct bandgap of 1.26 eV, which is solely attributable to the interaction between the Fe and O atoms within the magnetite cluster. Moreover, Figure 4b shows that overcoming this potential energy barrier enables a transition from spin up to spin down. Figure 4c displays the TDOS for the magnetite cluster. Notably, it reveals the presence of both spin polarizations at the Fermi level, with the spin-down polarization being the dominant one. This observation is indicative of a ferromagnetic behavior, aligning with the findings of Ermakov and colleagues [56]. Furthermore, an initial bandgap of ≈0.68 eV is observed, leading to a spin-down state, and an additional energy of ≈0.2 eV is needed for the transition to a spin-up state. Figure 5 illustrates the spin density difference (up–down) isosurface, with a value set at 0.01 *e*/Bohr^3^. This calculation considers only the valence states. The computed values of the difference fall within the range of −0.009 to 1.794 *e*/Bohr^3^, indicating that the effective component corresponds solely to spin up, consistent with observations in Figure 4b. Furthermore, the figure demonstrates that the spin density difference is practically associated with the cluster. The studies also revealed that the total magnetic moment of the cluster alone has a magnitude of 10 μB; when it is part of the composite, it reaches a value of 12 μB. These results show that the confinement effect of the NaA zeolite offers the possibility of altering not only the electronic and magnetic properties, but also the spin channels of the cluster.

**Figure 6:**
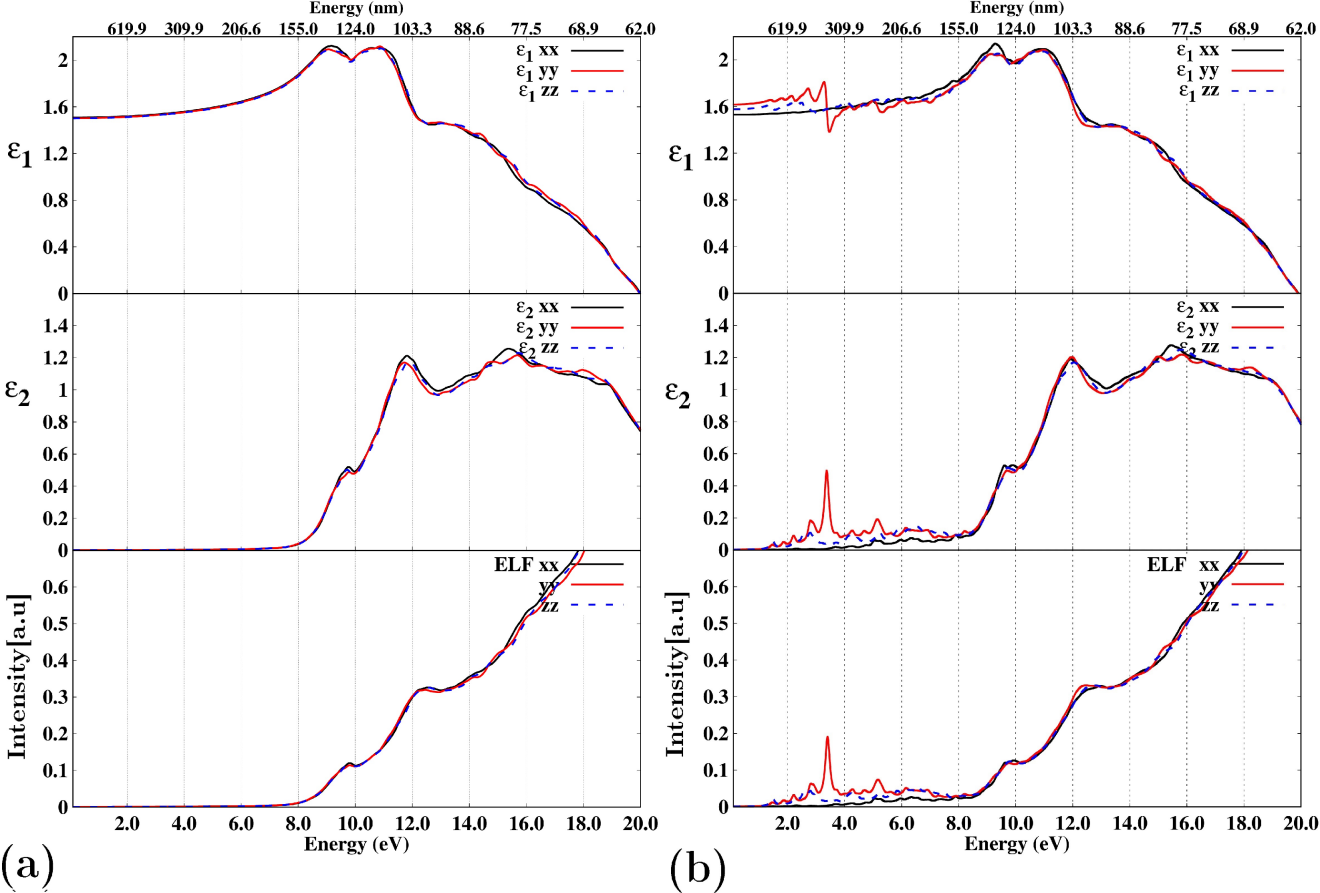
Real and imaginary parts of ε and ELF for (a) NaA zeolite and (b) NaA-M composite. The energy range is given in both energy (eV) and wavelength (nm). The curves labeled *xx*, *yy*, and *zz* correspond to the diagonal components of the tensors for both the relative permittivity and the ELF.

Figure 6 provides a comparison of the behavior of the real and imaginary components of relative permittivity ε(=ε_1_ + *i*ε_2_) and energy loss function (ELF) for the zeolite NaA (Figure 6a) and the NaA-M composite (Figure 6b). Although both the relative permittivity and the ELF are described by a 3 × 3 tensor, for simplicity, we will focus solely on the dominant components located on its main diagonal, labeled as *xx*, *yy*, and *zz*, respectively. In Figure 6a, it is evident that for energies below 6.8 eV (the value that corresponds to the zeolite bandgap), the value of ε_2_ is practically zero. Under such circumstances, the velocity (*v*) of electromagnetic wave propagation in a dielectric medium can be described as 
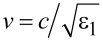
, where *c* is the speed of light in a vacuum. Within this range ε_1_*xx* = ε_1_*yy* = ε_1_*zz* then ε_1_ = 1.5; consequently, the speed with which an electromagnetic wave propagates through this zeolite is *v* = 0.81*c*. Of course, the speed of propagation of electromagnetic waves outside this range may not be the same. Given that ε_2_ quantifies energy dissipation within the medium [64], Figure 6a shows that ideally the zeolite exhibits null dissipation within this energy range. Figure 6a indicates that the zeolite exhibits negligible dissipation in this specific energy range. Particularly within the visible spectrum (ranging from 1.63 to 3.26 eV or from 380 to 700 nm), this absence of dissipation shows that the material is transparent, a characteristic commonly associated with distinct aluminosilicates [65–73]. Given that the ELF is connected to the relative permittivity as follows [74]:


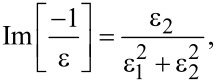


it is anticipated that its behavior below the bandgap value will be consistent with that observed in Figure 6a.

Figure 6b shows that when the cluster is introduced into the zeolite, the relative permittivity of the NaA-M composite exhibits a certain degree of anisotropy. In particular, for the range from 0 to 8 eV, the component ε_1_*yy*, both real and complex, has some intervals where it is the highest. Note that within this interval is the visible range; therefore, unlike pure zeolite, the composite exhibits a response within the visible and ultraviolet regions of the spectrum (i.e., non-zero dissipation). For energies less than 1.26 eV, all imaginary parts of the relative permittivity are zero. In this small range, for an average value of the relative permittivity, ε_1_ = 1.6, we obtain that *v* = 0.79*c*, which means that within this energy range, electromagnetic waves slow down by about ≈2.5% compared with pure zeolite.

**Figure 7:**
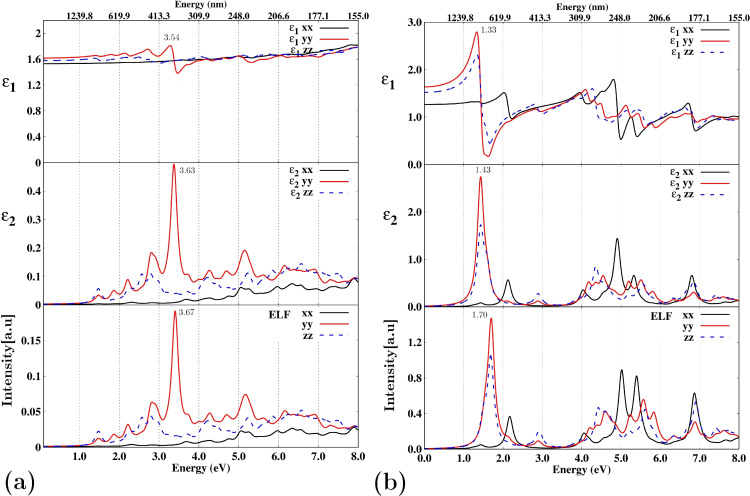
Real and imaginary parts of ε and ELF for (a) NaA-M composite and (b) isolated magnetite cluster. The energy range is given in both energy (eV) and wavelength (nm). The curves labeled *xx*, *yy*, and *zz* correspond to the diagonal components of the tensors for both the relative permittivity and the ELF.

In Figure 7, we compare the behavior of the real and imaginary components of the relative permittivity, as well as the ELF, in the energy range from 0 to 8 eV when the cluster is part of the NaA-M composite and when it is isolated. A direct comparison of the optical properties between these two scenarios reveals significant differences. For instance, in Figure 7a, it is evident that the primary peak of ε_1_ occurs at 3.54 eV, while for ε_2_ and the ELF, the peak energies are very close to each other (3.63 and 3.67 eV, respectively). In contrast, for the isolated cluster (Figure 7b), the positions of the main peaks in ε_1_, ε_2_, and the ELF exhibit notable differences. Additionally, it is noteworthy that the ELF exhibited by the composite closely mirrors the behavior of ε_2_, whereas, for the cluster alone, ε_2_ and the ELF demonstrate substantial discrepancies within the 4.5 eV to 6.0 eV range.

These results indicate that, for the composite, it is possible to deduce ε_2_ from the ELF, which is not valid for an isolated cluster. Thus, it becomes evident that the optical response of the magnetite cluster within the zeolite differs from its behavior when existing in isolation. An additional observation worth noting is the behavior of both ε_2_ and the ELF of the composite (as shown in Figure 7a), which indicates that, unfortunately, the primary peak occurs outside the visible range. However, considering that the current results highlight the modification of the cluster’s optical properties when introduced into the zeolite, there is potential to stimulate a peak response within the visible spectrum. This could be achieved by either adjusting the Si/Al ratio of the zeolite framework or by exploring the possibility of introducing the cluster into a different zeolite structure. These approaches offer avenues for tailoring the optical characteristics for specific applications within the visible spectrum.

## Conclusion

In this work, the influence of the inclusion of a magnetite cluster into NaA zeolite is studied through DFT calculations. The findings reveal that the cluster not only introduces states into the forbidden energy gap of the zeolite, but it also affects the band structure of the zeolite framework. Additionally, the geometry of the cluster stabilized in the zeolite cavity undergoes structural changes, which leads to modifications of its electronic and magnetic properties. Specifically, the investigation shows that the cluster within the zeolite exhibits characteristics of a half-semiconductor in contrast to the free cluster in the vacuum, which presents ferromagnetic behavior. Moreover, the results suggest that introducing the cluster into zeolite enhances the control over the transition between spin polarizations, making it a promising avenue for further exploration in spin-related applications.

The examination of optical properties reveals that including a magnetite cluster in the zeolite gives it an optical response within the visible and ultraviolet range of the spectrum. It should be noted that the optical properties exhibited by the cluster within the zeolite differ from the optical properties of the cluster outside the zeolite. This suggests that zeolites, when combined with certain clusters, can be effectively utilized to achieve an optically desirable response, particularly within the visible region of the spectrum.

## Data Availability

The data that supports the findings of this study is available from the corresponding author upon reasonable request.
